# Australian agricultural resources: A national scale land capability map

**DOI:** 10.1016/j.dib.2022.108852

**Published:** 2022-12-24

**Authors:** Vanessa M. Adams, Jayden E. Engert

**Affiliations:** a School of Geography, Planning, and Spatial Sciences, University of Tasmania, Australia 7001; b Centre for Tropical Environmental and Sustainability Science, James Cook University, Australia 4878

**Keywords:** Agricultural land capability, Land capacity, Land suitability, Cropping, Horticulture, Forestry, Grazing

## Abstract

Ongoing land clearing is a key driver of biodiversity loss and climate change. Effective action to halt land clearing and land degradation ultimately relies on understanding patterns of land capability for production uses, in particular agriculture, as a key driver of land use. Here we describe a national agricultural land capability map for Australia, based on harmonized state agricultural land capability datasets and modelled pastoral capability. State-level agricultural land capability datasets capture regional variations in crop selection and suitability. Hence, we reclassified these datasets to fit a nationally consistent land capability ranking scheme. For regions in which agricultural capability data was not available, we modelled agricultural and pastoral capability and mapped this to the same ranking scheme. The national land capability dataset fills an immediate knowledge need for Australia. This dataset has wide potential for utilization, such as for retrospective analysis of land use policies and prospective regional planning initiatives to ensure forward looking policies and land use plans optimize land allocation.

**Specifications Table**

**Table:** 

Subject	Soil Science, Agriculture, Nature and Landscape Conservation
Specific subject area	Nature and Landscape
Type of data	GeoTiff
How the data were acquired	This data record contains a national agricultural land capability map for Australia, based on existing state agricultural land capability datasets reclassified to a single agricultural land capability classification (NSW method) and modeled grazing suitability. Where data were missing land capability was modelled based on available spatial data inputs.
Data format	Raw
Description of data collection	The data includes three raster layers. The national scale agricultural land capability map is the primary data product available in the agricultural land capability data package (raster layer with 9 classes adhering to the NSW land capability mapping method definitions) (provided as land_capability GeoTIFF). The compiled state and territory land capability data on the unified rating class system is available as a sub product of the agricultural land capability data package (existing_capability GeoTIFF). Pastoral capability based upon modeled grazing suitability is available as a sub product of the agricultural land capability data package (pastoral_capability GeoTIFF).
Data source location	Australia
Data accessibility	Name of the repository: University of Tasmania Research Data Platform Title of the dataset: Mapped Agricultural Land Capability Classification Data for Australia V.20. Direct (URL) link to the dataset: https://dx.doi.org/10.25959/kjdc-k508 Data identification number associated with this dataset: doi:10.25959/kjdc-k508.

## Value of the Data

• Given agriculture is a primary global land use, and maintaining agricultural production is an imperative for food security, understanding agricultural land capability is key to land use planning to ensure both continued provision of food production while also halting land degradation to protect nature.
• Around 15% of Australia's total land area has been cleared for agricultural or productive land uses, while only ∼0.5% has been converted for urban areas, rural residential areas, and waste and mining uses combined [1]. Consistent agricultural land capability mapping is thus a critical tool in Australia to inform land use planning and improve land use decisions.
• National scale agricultural land capability data is useful for scientists, planners, conservation organisations, and other organisations making decisions regarding nature conservation and land use policies in Australia.
• This national scale data set is expected to create important opportunities for spatial analysis of diverse questions such as: where is land likely to be cleared for agricultural land uses into the future and therefore what areas of Australia are most at risk of future habitat loss, how can regional planning best guide conservation and development to ensure best use of land resources, and to what extent have Australia's national protected areas been designed to avoid habitat loss or conversely to what extent are they residual in nature.

## 1 Objective

Land capability is a classification system that ranks land according to the capability to support agricultural production broadly, based on various uses such as broadscale grazing and cropping. Land capability is primarily determined by underlying biophysical characteristics (e.g., geology, soil, slope, and climate) and physical limitations (e.g., drainage, flooding, erosion hazard) [2], [3], [4], [5]. Land capability differs from other related measures of landscape productivity such as land condition (current state or quality of soil resources), suitability (ability to sustain a particular land use such as a single cropping type) [6], or versatility (ability of land to support versatile uses) [7].

Land capability mapping has a long tradition of being used as a key data product to guide land use planning [3,^5^,8] and optimal placement of agricultural businesses [9], as well as identifying where land clearing is happening on inappropriate lands and halting future land clearing and degradation by using land clearing policies or protected areas to counter habitat loss [10,11]. Despite the utility of national scale land capability mapping, no consistent map of agricultural land capability exists for Australia. To fill this data gap, we introduce here the national Australian map of agricultural land capability which draws together jurisdictional level maps based on a single classification and creates and combines with a national pastoral capability layer to fill remaining spatial gaps in data.

## 2 Data Description

**Fig. 1:**
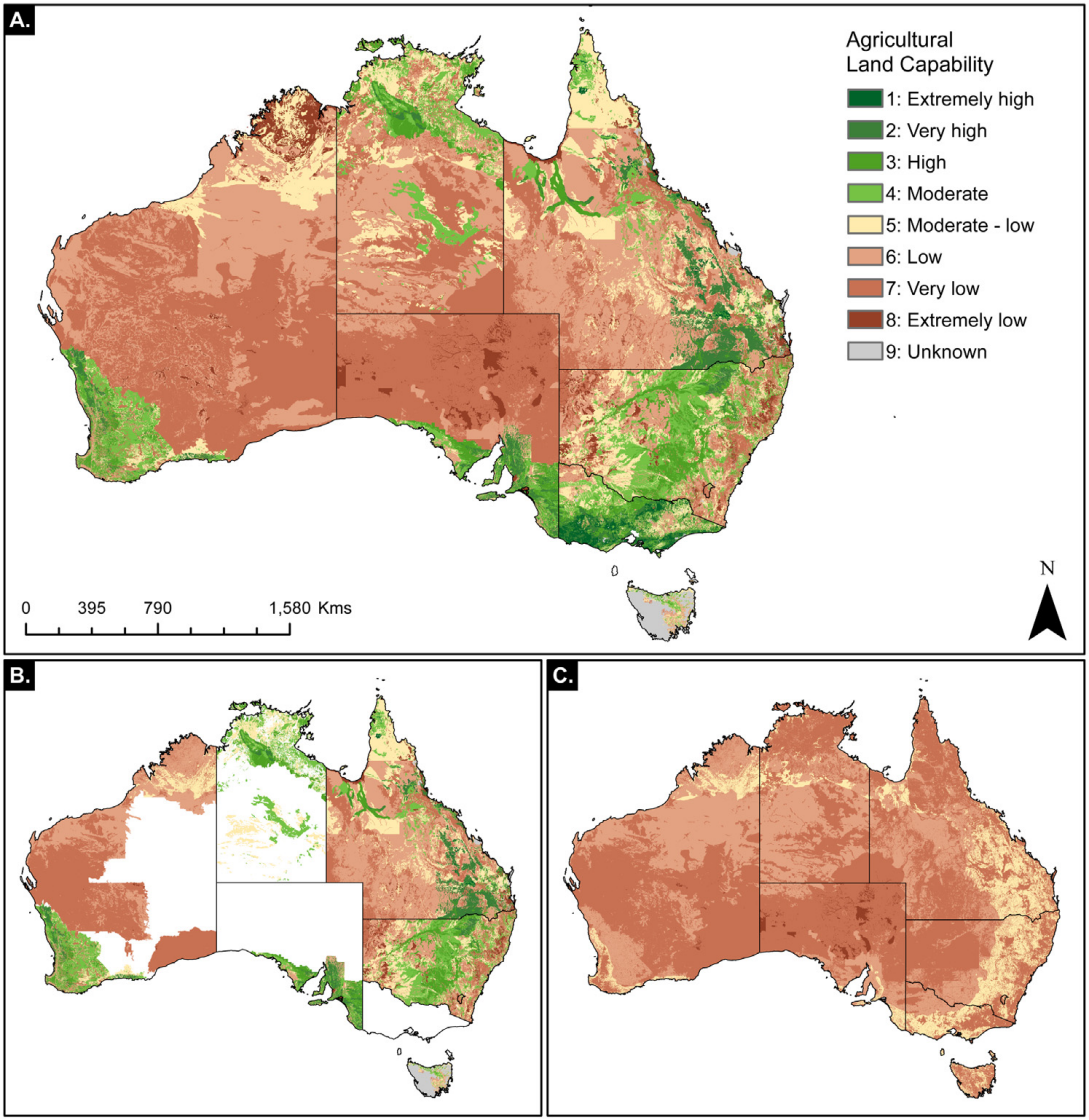
Map of land capability for supporting agricultural production land uses across Australia. A) Full land capability data. B) Existing state level data compiled and harmonized to the chosen land capability rating method. C) Australia-wide pastoral capacity layer harmonized to the chosen land capability rating method.

A map of the final national agricultural land capability dataset and intermediate data outputs is provided in Fig. 1. The land capability data adheres to a rating system developed and applied in New South Wales (NSW) Australia that rates land capability from 1 – 9 [12]. The mapped data can be interpreted using the definitions provided in Table 1.

**Table 1:** Land capability class definitions and key characteristics. These definitions and criteria were used to guide the unification and mapping of all other jurisdictions methods to the 9 classes.

Rating Class	Definition	Limitations	Choice of crops	Required management practices
Land capable of a wide variety of land uses (cropping, grazing, horticulture, forestry, nature conservation) and typically under cultivation				

1	Extremely high capability land: Land has no limitations. No special land management practices required. Land capable of all rural land uses and land management practices	None to Very minor	Any	None to Very minor

2	Very high capability land: Land has slight limitations. These can be managed by readily available management practices. Land capable of most land uses and land management practices.	Slight	Slightly reduced	Minor

3	High capability land: Land has moderate limitations. These can be managed by readily available management practices. Land capable of most land uses and land management practices.	Medium	Reduced	Major

4	Moderate land capability: Land has moderate to high limitations for high-impact land uses. Will restrict land management options for high impact land uses such as cropping, horticulture, and high-intensity grazing which can only be managed by specialised management practices with high level of knowledge, investment and technology.	Medium to High	Restricted	Major + Careful management

Land capable for a limited set of land uses (grazing, forestry, some horticulture, and nature conservation) and typically under pastoral use				

5	Moderate - Low capability land: Land has very high limitations for high impact land uses. Will largely restrict land use to grazing with potential for some horticulture (orchards) and forestry. Limitations need to be carefully managed to prevent long-term degradation.	High	Grazing	Major + Careful management

6	Low capability land: Land has very high limitations for high impact land uses. Land uses restricted to grazing, forestry and nature conservation. Careful management of limitations required to prevent severe land and environmental degradation	Very high	Grazing	Major + Careful management

Land generally incapable of agricultural land uses (selective forestry and nature conservation)				

7	Very low capability land: Land has severe limitations for most land uses and generally cannot be overcome. Impacts of land management practices can be extremely severe if limitations not managed. There should be minimal disturbance of native vegetation.	Very severe to extreme	No, or very minor agricultural value	Major + Careful management

8	Extremely low capability land: Limitations are so severe that the land is incapable of sustaining any land use apart from nature conservation. There should be no disturbance of native vegetation.	Extreme	No agricultural value	

9	Excluded from mapping	N/A	N/A	N/A

The spatially referenced national scale GeoTIFF data package is available for download from the University of Tasmania Research data portal: https://dx.doi.org/10.25959/kjdc-k508.

The three national scale layers included in this data package are:

land_capability GeoTIFF: Australia-wide agricultural land capability data is the primary product in the data package as displayed in Fig. 1A. The data is in spatial reference GCS GDA 1994 at a 0.0003 decimal degree resolution.
existing_capability GeoTIFF: Existing state and territory level data compiled and harmonized to the unified rating class system is available as a sub product of the agricultural land capability data package as displayed in Fig. 1B. The data is in spatial reference GCS GDA 1994 at a 0.0003 decimal degree resolution.
pastoral_capability GeoTIFF: Australia-wide pastoral capability based upon modeled grazing suitability is available as a sub product of the agricultural land capability data package as displayed in Fig. 1C. The data is in spatial reference GCS GDA 1994 at a 0.0003 decimal degree resolution.

## 3 Experimental Design, Materials and Methods

Here we define agricultural land capability as the capability of land to support a range of agricultural land uses and adopt a classification approach consistent with international classification schemes using a systematic arrangement of different kinds of land according to those properties that determine the ability of the land to produce permanently [5]. We note that our methods here specifically adopt a land capability definition and appropriate set of methods for mapping agricultural and pastoral land capability [2], [3], [4], [5].

The national context of land use, appropriate crops and livestock mixes, and history of production are critical for informing meaningful land capability maps. We therefore chose to focus on Australia-specific land capability mapping methods [12], but note these are closely related to and derivatives of the USDA [4] land capability mapping methods which have also been used to inform national scale approaches such as in the United Kingdom [3].

**Fig. 2:**
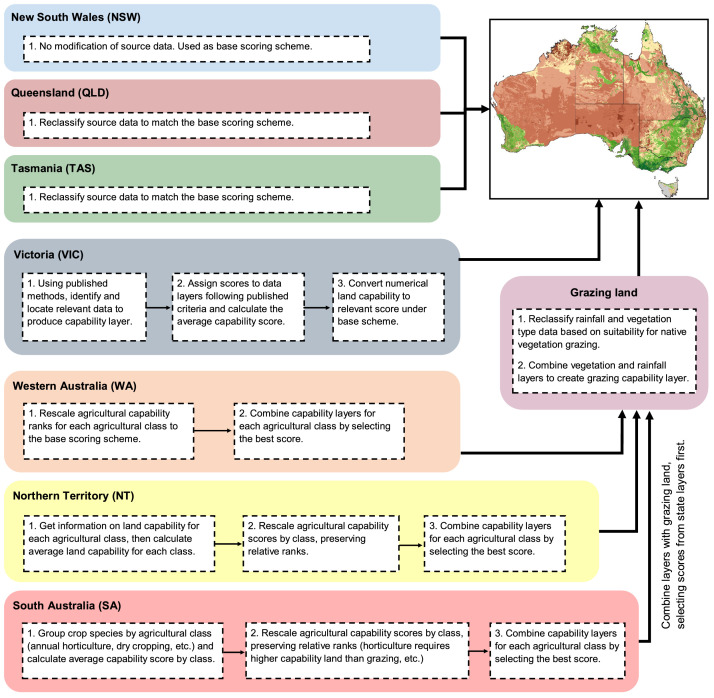
Flow chart for methods at jurisdictional level for mapping agricultural land capability. Type 1 jurisdictions (NSW, QLD, TAS) required reclassification. Type 2 jurisdictions (WA, NT, SA) involved rescaling and combining multiple layers and then combining with modelled grazing capability to fill remaining gaps. Type 3 (Vic) required complete mapping based on published methods and available input data.

Our method included first reviewing the jurisdiction level methods and mapping products to inform a final choice in method and land capability rating. We then compiled all available jurisdiction level mapped land capability products. Because jurisdictions often have finer resolution data and improved interpretation of land capability, we chose to use original jurisdiction data products where possible and to harmonize these to a uniform classification. Where jurisdictions did not have any mapped land capability data or incomplete land capability data, we modelled land capability using jurisdictional level data inputs and based upon our final chosen classification method. Lastly, we produced a national scale pastoral land capability data product which was used to fill any remaining gaps in mapped agricultural land capability. We outline each of these steps in Fig. 2 and in detail below.

### 3.1 Agricultural land capability methods

A review of the land capability assessment methods used across Australian jurisdictions, as well as completeness of available mapping, demonstrated that a very similar rating method was used across New South Wales (NSW) [12], Queensland (QLD) [13], and Tasmania (TAS) [14]. New South Wales presented the most recently updated and comprehensive method and map out of these three jurisdictions. Thus, New South Wales class definitions were used as the base method by which to adapt methods from all other state and territories [12]. The rating class definitions and key characteristics (limitations, choice of crops, and required land management practices) as used here (termed from here on out as the ‘rating scale’) are provided in Table 1.

#### 3.1.1 Jurisdiction level agricultural land capability mapping

We compiled all available state and territory land capability data sets, reviewed them against our land capability rating, and then manipulated them to match our land capability rating. Manipulations of agricultural land capability datasets at the state and territory level fell into three main methodological forms: (1) reclassification to fit the agricultural capability selected rating scale, (2) integration of multiple land capability maps and reclassification to fit the agricultural capability rating scale, and (3) creation of new land capability map following existing guidelines. Queensland and Tasmania both had existing comprehensive land capability datasets which were reclassified to fit the New South Wales rating scale (type 1). Western Australia (WA), South Australia (SA), and the Northern Territory (NT) had existing land capability maps for various different land use types (i.e. horticulture, field cropping, grazing) which were integrated into a single comprehensive dataset before being reclassified to fit the New South Wales rating scale (type 2). Finally, Victoria (VIC) had no existing land capability dataset and hence it was necessary to create a new dataset (type 3).

Existing land capability datasets for Queensland and Tasmania were reclassified to the selected rating scale following criteria outlined in Table 2. Reclassifications were largely one-to-one, save for classes ‘C’ and ‘C1’ in the Queensland scale being grouped together for conversion to the New South Wales scale, and the absence of an equivalent to class 8 in the Tasmania dataset.

**Table 2:** Individual state and territory land capability classes mapped to the NSW rating used in this study.

Rating Class	NSW	QLD	VIC	SA	WA	TAS	NT
1	1	‘A’	1	—	A1	1	—

2	2	‘A1’	2	Aa, Ab	A2	2	2

3	3	‘A2’	3	Ac, Ad	B1	3	3

4	4	‘B’	4	B	B2	4	4

5	5	‘C’ and ‘C1’	5	C	C1 or <4ha/cu in rangelands grazing cap	5	5

6	6	‘C2’	6	D	C2 or 4-100ha/cu rangelands grazing gap	6	N/A

7	7	‘C3’	7	Ea	>100ha/cu rangelands grazing cap	7	N/A

8	8	‘D’	8	Eb	unsuitable' rangelands grazing cap	N/A	N/A

9	N/A	‘Unknown’	N/A	X	N/A	E	

For Western Australia [15], South Australia [16], and the Northern Territory [17], existing information on land capability was divided into various land use classes (Table 2). For these cases, we rescaled each separate existing dataset to fit the selected rating scale individually and then integrated the land capability datasets. As different land use types have different requirements, each land capability dataset was reclassified based on both the land use type, and the capability of supporting said land use. For example, land with moderate capability to support grazing often has low capability to support annual horticulture. The full details on existing land capability information and the reclassification methods for Western Australia, South Australia, and the Northern Territory are outlined in the appendix.

While Victoria did not have an existing land capability dataset, they did have a published method [18] that could readily be unified with the 9-class system selected. Thus, we created this map at a jurisdiction level by following, as much as possible, their published method and using state-wide available data (see appendix for full details). In cases where information outlined in the published methods was not available, we substituted available information deemed to be equivalent or similar, i.e. replacement of information on soil depth with information on structure of subsoil.

The compiled state and territory land capability data on the unified rating class system is available as a sub product of the agricultural land capability data package.

#### 3.1.2 Pastoral capability method

There were large spatial gaps in the available mapped land capability for Western Australia, South Australia, and the Northern Territory. These gaps were primarily in the arid zone of states where agricultural land use is primarily native vegetation grazing. The existing spatial products focused on areas suitable for cropping and thus did not map areas that would be largely constrained to grazing. Therefore, to fill this gap in the spatial data we modelled pastoral capability for the remaining areas (all mainland states and territories restricted to grazing focused land capability classes 5-8, Table 1).

Based on published reports on grazing land capacity [19,20] we defined pastoral capability as a function of vegetation type and mean annual rainfall (see Table 3 for data sources). Vegetation and rainfall were each independently classified in terms of relative grazing capability. Vegetation was classified as either not conducive to pasture (highly modified classes, woody classes and water, class = 0), or conducive (all other types, class = 1). Mean rainfall was classified as a value of 2 (areas capable of supporting high value pasture) for annual rainfall greater than 500mm, 1 (areas capable of supporting low value pasture) for annual rainfall of 200-500mm, or 0 (capable of supporting only very low pastures) for rainfall less than 200mm. Vegetation and rainfall classes were then combined (using addition function in GIS) to produce a pastoral capability layer with the values of 0 = not conducive to pasture (score of 8 based in unified rating system); 1 = minimal use for pasture (score of 7 based in unified rating system); 2 = low value land for pasture (score of 6 based in unified rating system); 3 = moderate to high value pasture land (score of 5 based in unified rating system). The pastoral capability layer is available as a sub product of the agricultural land capability data package with classes 0 to 3. Lastly, we classified the pastoral capability layer into the unified rating system to values of 5-8 in order to be able to mosaic with jurisdiction data for a unified national scale agricultural land capability dataset.

**Table 3:** Summary of existing data sets used and their describing resolution and limitations.

	Resolution	Limitations	Data reference
Australia wide (pastoral capability inputs)			

Rainfall	0.05 degree grid cells	Mean monthly and mean annual high resolution rainfall grids. The grids show the rainfall values across Australia in the form of two-dimensional array data. Climatological period 1981-2010. Values estimated by positional interpolation from weather station locations.	BoM. 2020 [25]

Vegetation	100m Grid Cells	Input datasets dated between 2006 and 2009. Positional accuracy dependent on the input dataset.	DoEE. 2017 [26]

State and territories (land capability products)			

NSW	Resolution varies with region, ranges from 1:100,000 along the coast to 1:500,000 in the western areas. A Soil Data Confidence Map is available as metadata.	The LSC assessment scheme is most suitable for broad-scale assessment. The LSC assessment scheme is less suitable for high intensity land use or for irrigation than it is for low-intensity use, dry-land agriculture uses.	OEH. 2012 [12]

QLD	∼25% of the area of the state is at a resolution of 1:250,000 or better, the rest is between 1:500,000 and 1:1,000,000.	Not designed to be suitable for localised planning. 2012-2013 Audit was desktop based with data inputs ranging in resolution from 1:50,000 to 1:2,000,000. 86% of the state has low land resource data confidence.	DAFF. 2013 [13]

VIC	Resolution of input data layers ranged from 10m x 10m grid to 1:250,000 vector format.	Reliability of information varies across the region. Land capability map generated by the report author from available data rather than a dedicated Natural Resource Management authority (see appendix for further data input details).	Rowe et al. 1981[18]

SA	Default scale of 1:100,000 with some regions of more intense land use being mapped at 1:50,000.	Based on limited data and have not been subjected to field validation. Models are based on soil and landscape properties alone (climate factors not considered). Mapping data is a simplified version of the Analysis data produced by the report.	Rowland et al 2016 [16]

WA	Soil-landscape mapping was at scales between 1:25,000 and 1:250,000.	Land capability estimated using limited available information, has not been subject to field validation. Land capability based on soil and landform attributes, no climate information.	van Gool et al 2005 [15]

NT	Northern land systems mapped at 1:250,000. Southern land systems mapped at 1:1,000,000. Groundwater assessment is based on 1:2,000,000 dataset.	Low resolution of datasets. Groundwater dataset only takes into account availability, not quality (salinity etc).	Pascoe-Bell et al 2011 [17]

TAS	Default scale of 1:100,000	Very limited field checking has been undertaken yet the maps are considered to provide a reliable representation of land capability	Groese 1992 [14]

#### 3.1.3 Compiling the final national agricultural land capability map

We mosaicked together the agricultural land capability maps based on the jurisdiction level mapped data and our national scale pastoral capability layer to create a final comprehensive land capability map. The national scale land capability map is the primary data product available in the agricultural land capability data package. A map of the final national agricultural land capability dataset and intermediate data outputs is provided in Fig. 1.

Sources of data that have been used in the creation of the dataset are listed in Table 3.

### 3.2 Technical Validation

#### 3.2.1 Description of limitations and sources of error

The primary sources of data limitations and error are from the original jurisdictional data and national scale inputs for pastoral capability. Thus, we have summarized all documented limitations to each jurisdictional land capability map and data inputs for pastoral capability in Table 3.

#### 3.2.3 Validation tests

We note that ideally ground truthing data points would be collected to test validity of the modelled land capability. However, for a national scale dataset that focused on using jurisdiction level data inputs that have individually been validated that this was not feasible. Instead, we report on the usage notes and issues associated with each of those products as a guide for likely error and validity. We further test the robustness of our land capability classification method through other tests including validation across jurisdiction borders and relationship between land capability and land use mapping products.

Given our methodology was built to use jurisdictional data where possible, we first wished to validate the extent to which jurisdictions were uniformly applying the national land capability mapping standards. If there are variances in jurisdictional interpretation of methods or input data quality, we would expect there to be more variation in the classification of adjoining pixels at the jurisdiction borders. Conversely, if jurisdictions are using input data of similar types of quality and following the national standards then we should not find variation in classes of adjoining pixels at jurisdiction borders. Thus, we first tested classification agreement at state and territory borders.

Second, we tested the correlation between agricultural capability, land use, and land clearing to validate that our dataset does reflect that land capability classes largely match the types of agricultural land uses mapping to particular classes (aligned with the appropriate uses described in the classification). Land holders make rationale land use decisions based on the underlying capability of agricultural land. We thus would expect for there to be a strong, near one to one, relationship between agricultural land capability and agricultural land use particularly in a nation such as Australia which has engaged in agricultural land uses since colonisation. Second, land capability datasets are produced by state and territory governments in order to facilitate efficient utilization of lands for productive land-use types, such as agricultural land or pastoral land. This is particularly clear in states such as South Australia and Western Australia where only specific regions deemed to be capable of supporting productive land uses have been mapped. This indicates that productive land uses should largely be confined to areas with high ranks in land capability as a basis of land use policies. Hence, comparing overlap of productive land-use classes with land capability can be used to validate the accuracy of our dataset. Furthermore, land capability should dictate the land uses and thus also the extent to which land is cleared on individual properties (assuming producers have knowledge of the land capability and are making rationale choices to clear and produce on high capability land first). Comparing our mapped capability to patterns of clearing provides further validation of our dataset.

##### 3.2.3.1 Border analysis

The Australia-wide dataset combines maps with varying levels of spatial accuracy, resolution, and study effort. As such, some bioregions or geographic regions may score differently in different areas if they cross state boundaries, or into regions under differing study effort. For example, the Mulga Lands bioregion spans northern NSW and southwest Queensland. While this bioregion is predominantly semi-arid to arid it has been historically used for native grazing and improved pasture grazing alongside other agricultural uses. The soil and climate characteristics are consistent across the state boundaries and thus we would expect the classification to be uniform at the state border.

To test for agreement of our Australia-wide dataset across state borders we calculate the standard deviation for land capability across a focal range. Due to variation in overlap ranges between state and territory maps, our focal range spanned a 1km buffer either side of each state/territory border. Hence, focal statistics (standard deviation) were calculated for a window of 1 cell x 2km for the length of each border. Using this method, we calculated a focal window standard deviation for each map cell at jurisdictional borders.

**Fig. 3:**
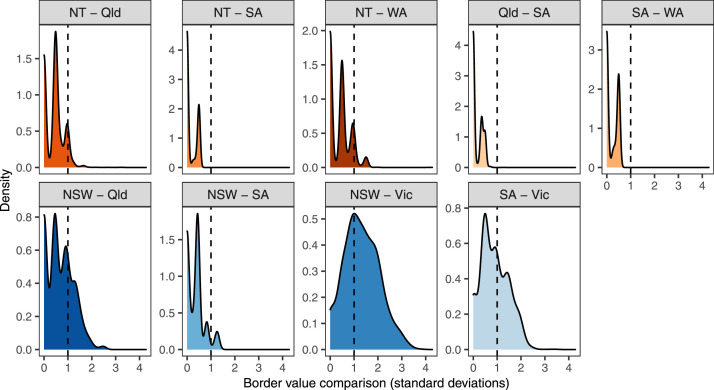
Comparison of land capability values around state and territory borders. Density plots in warmer colours indicate border comparisons in the inland part of the country where land capability was often lower and less variable at small spatial scales. Density plots in cooler colours indicate border comparisons closer to coastal areas and areas where land capability was more variable at small spatial scales. Darker coloured plots indicate longer borders.

Finally, to compare border agreement of the Australia-wide land capability dataset, we calculated the density of standard deviation values across the whole border for each jurisdiction border (Fig. 3). In this figure, plots with higher density of low standard deviation values (below 1) demonstrate greater agreement across the state/territory border. The vast majority of focal window statistics, and the majority of borders, show high agreement thus validating our approach of using jurisdictional data as the primary data source. The borders with the largest standard deviation were NSW and QLD, NSW and VIC, and SA and VIC (Fig. 3). The NSW-QLD variance is notable given each jurisdiction methods are very similar in nature and the bioregions that cross state borders have similar characteristics and land use patterns. This variation could indicate variability in human interpretation of classifications. The variation between NSW and VIC, and SA and VIC, likely reflects variation in the resolution or quality of data inputs given we created Victorian dataset following Victoria's published methods using newer and higher resolution data.

Given state level mapping is likely to include local nuances and improved data inputs, we chose to retain state data where available without any smoothing of classifications at jurisdiction borders. However, we note that users can choose to smooth jurisdictional boundaries in particular using our pastoral capability data layer.

##### 3.2.3.2 Land capability against land use classes

Based on the classification definitions and rationale landholder choices to use best available land resources for production, land capability classes should map closely to observed land uses. Lands in agricultural classes 1-4 would be expected to primarily be in agricultural uses including all types of cropping. Additionally, as some pastoral land uses have potential for high profitability (i.e. dairy cattle) we would expect some amount of pastoral land on classes 1-4. Land in classes 5-6 is generally unsuitable for intensive agriculture and would be expected to be used for grazing and forestry. Land in class 7 is expected to be restricted to use for low intensity production such as native vegetation grazing and forestry, or non-productive land uses such as conservation. Land in class 8 is unsuitable for any productive land uses and is expected to be primarily intact vegetation. To test this, we overlaid land capability classes against mapped land use from the Australian Collaborative Land Use and Management Program ​(ACLUMP​) [1].

**Fig. 4:**
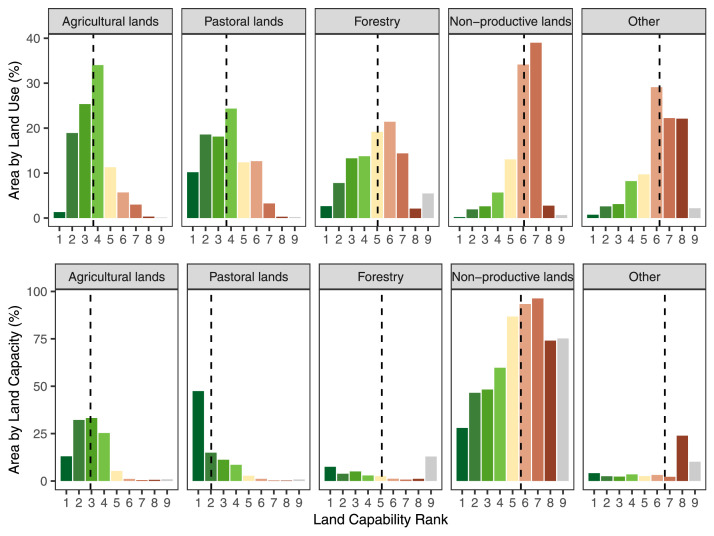
Area by land capability for major land use categories. First row shows land capability scores as a percentage of each land use class. Second row shows land capability scores as a percentage of all land of that capability score. Colours of bars match land capability scores in mapping product. Dashed line indicates the mean value for each land use.

We found that the vast majority of land currently utilised for agriculture or pastoral uses scored high in land capability from our land capability dataset (Fig. 4). Similarly, a large portion of the land in Australia scoring high in our land capability index was under some form of productive land use. Non-productive land-uses, including nature conservation, were largely confined to lands scoring low in our land capability index. This validates that our land agricultural capability map is accurate insofar that it meets our expectations that it should be an underlying driver of existing land use patterns.

##### 3.2.3.3 Land capability against land clearing rates

Areas with high land capability were often utilised for productive land-uses (Fig. 4). Hence, we should find that land capability has strong association with land clearing rates. To test this assumption, we used the National Vegetation Inventory System (NVIS) present theme data for 2020, to identify cleared or non-native vegetation [21]. In order to identify the relationship between land capability and land clearing we used a generalized linear model on a stratified random sample of the Australia-wide map. To ensure our modelled correlations were not subject to spatial autocorrelation, we took a stratified random sample of the agricultural capacity map (stratified by agricultural capacity scores) that retained 49,704 cells evenly distributed between capacity classes. We confirmed that our sampled sites were not subjected to high levels of spatial autocorrelation by calculating Moran's I using the moranfast package in R [22]. While the level of spatial autocorrelation present in the sample was statistically significant, the effect size was very low, and hence we did not consider it to be influential (I = 0.040, p = 0).

We modelled the relationship between land capability and land clearing using a binomial GLM, with cleared land having a value of 0 and remnant native vegetation having a value of 1. Model performance was assessed using the DHARMa package in R [23] (Hartig, 2022). Additionally, we compared the probability of being cleared between different land capability values by calculating estimated marginal means using the package ‘emmeans’ [24]. Finally, we ensured our stratified random sample was representative of the Australia-wide map by comparing model probabilities against the complete dataset.

**Fig. 5:**
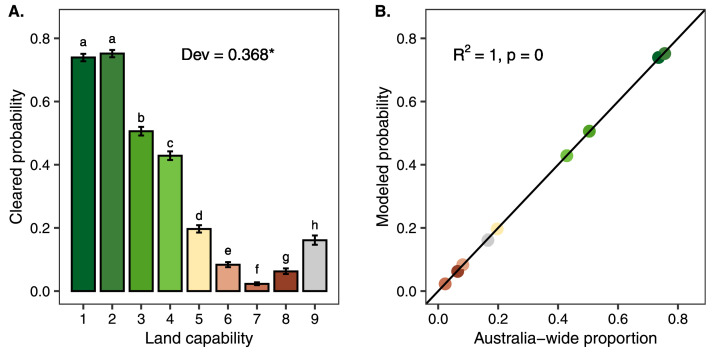
Land capability and land clearing statistical relationship. A) Clearing probability as a function of land capability. Letters above bars indicate capability scores that differed in modelled clearing rates. Dev indicates the proportion of deviance in land clearing explained by land capability alone with the asterisk denoting a significant relationship. B) Modelled probability compared to Australia-wide proportion to validate model sample. Overall our model demonstrates that land capability adheres to the expectation that it is a strong predictor of land clearing or conversion to non-native vegetation.

We found that land capability was a strong driver of land clearing, with our model containing only land capability explaining 36.8% of the deviance in which areas were cleared (Fig. 5A). Areas with higher land capability also had significantly higher clearing rates, with the exception of land in class 8 having a higher clearing rate than land in class 7. Land in class 8 is often in arid regions of the country where mining takes place, and hence some clearing of this class may be due to mine site development. Additionally, land in class 8 may be areas with a degree of topographic complexity that excludes agricultural uses but does not exclude removal of native vegetation for forestry purposes. The modelled probability of land clearing from our stratified random sample was perfectly correlated with the Australia-wide proportion of lands cleared by land capability class (Fig. 5B).

### 3.3 Usage notes

The agricultural land capability mapping product described in this paper can provide information relevant for land use planning to ensure the best use of limited land resources, halt further land degradation, and prioritise restoration to improve land condition across Australia. The data set harmonizes all state and territory agricultural land capability data to a single rating scheme, thus leveraging this data as the highest resolution validated data available but ensuring uniform interpretation and application. It further improves upon this data by filling gaps for agricultural and grazing land uses to provide a uniform mapping product across Australia. As such, it can be used for application at regional to national scale for the purposes of evaluating and guiding land clearing, nature conservation, and pastoral stewardship. Prospective planning can also leverage this dataset to ensure regional land use plans result in the best use of limited land resources to deliver on economic, social, and environmental goals.

## Ethics Statement

No ethics approvals were required or sought for this work.

## Code Availability

Data analyses were completed in ESRI ArcGIS and no code is supplied. Analyses can be reproduced following spatial analysis steps described in the methods.

## CRediT Author Statement

VMA conceived and led the study. VMA and JE collated the data. JE completed the analyses. VMA completed the first version of the manuscript and VMA and JE contributed to writing and editing the paper.

## Declaration of Competing Interest

The authors declare no potential conflicts of interest or competing financial interests

## Data Availability

Mapped Agricultural Land Capability Classification Data for Australia V.20 (Original data) (University of Tasmania Research data portal)

## Appendix Supplementary Materials

Image, application 1

## References

[1] ABARES (2016).

[2] MAFF (1988).

[3] Singer M.J. (2014). Land capability analysis. Encycloped. Natur. Resour. Land.

[4] USDA (1961).

[5] Wang Y. (2020).

[6] McBratney A.B., Field D., Morgan C.L.S., Huang J. (2019). On Soil Capability, Capacity, and Condition. Sustainability.

[7] Kidd D., Webb M., Malone B., Minasny B., McBratney A. (2015). Digital soil assessment of agricultural suitability, versatility and capital in Tasmania, Australia. Geoderma Regional.

[8] Akpoti K., Kabo-bah A.T., Zwart S.J. (2019). Review - Agricultural land suitability analysis: State-of-the-art and outlooks for integration of climate change analysis. Agric. Syst..

[9] Adams V.M., Pressey R.L., Álvarez-Romero J.G. (2016). Using optimal land-use scenarios to assess trade-offs between conservation, development, and social values. PLoS One.

[10] Adams V.M., Pressey R.L. (2014). Uncertainties around the implementation of a clearing-control policy in a unique catchment in Northern Australia: Exploring equity issues and balancing competing objectives. PLoS One.

[11] Brink B.J.E., Cantele M., Adams V.M., Bonn A., Davies J., Fernández M., Matthews N., Morris J., Ramírez Hernández W.A., Schoolenberg M.A., Berg M.v.d., Pennock D., Vuuren D.P.v., Montanarella L., Scholes R., Brainich A. (2018). The IPBES assessment report on land degradation and restoration.

[12] OEH (2012).

[13] DAFF (2013). http://www.daff.qld.gov.au.

[14] Grose C.J. (1992).

[15] van Gool D., Tille P.J., Moore G.A. (2005).

[16] Rowland J., Maschmedt D., Liddicoat C. (2016).

[17] Pascoe-Bell A., Lynch B., Hill J., Green C., Cameron A., Smith S. (2011).

[18] Rowe R., Howe D., Alley N. (1981).

[19] De Leeuw P.N., Tothill J.C. (1990).

[20] Holmes Sackett (2010). https://www.mla.com.au/contentassets/070d8da508f842d292aa7ae6a4c78d4e/b.com.0255b_final_report.pdf.

[21] Australian Government (2021).

[22] M. Cooper, moranfast: Conduct a Quick and Memory-Efficient Moran's I Test. R package version 1.0., 2020.

[23] F. Hartig, DHARMa: Residual Diagnostics for Hierarchical (Multi-Level /Mixed) Regression Models. R package version 0.4.5., 2022.

[24] R.V. Lenth, emmeans: Estimated Marginal Means, aka Least-Squares Means. R package version 1.7.2., 2022.

[25] BoM, Mean annual rainfall data, 2020 [dataset].

[26] DoEE, 2016 SoE Land Extent of all forms of vegetation across Australia, 2017 [dataset].

