# Supplementary material for: Australian agricultural resources: A national scale land capability map
Source: Data Brief. 2022 Dec 24;46:108852. doi: 10.1016/j.dib.2022.108852 (PMC9823136; doi:10.1016/j.dib.2022.108852)
Supplement: Supplementary file 1 [file mmc1.pdf]

Appendix

In this appendix we provide the detailed methods for how we interpreted jurisdictional data that had multiple datasets to compile these into a single classification that was analogous to the NSW rating [1]. This includes Victoria, Northern Territory, Western Australia, and South Australia.

Victoria detailed methods

Victoria had not generated land capability mapping layers. However, they had developed a Victoria Land Capability Assessment methodology [2]. The Layer Selection section describes the selection process for data to generate a land capability layer. The final land capability layer generated is a raster dataset with resolution of 270m x 270m with a 1 – 9 rating scale based on NSW rating system (same as Qld).

Layers were selected for a land capability map layer for Victoria using parameter information from their own land capability assessment methodology, as well as information about layers used in the land capability mapping for other states (QLD and NSW were the basis for layer selection).

Table S1 outlines parameters selected for use in generating the Victorian land capability layer. Parameters highlighted in green were used in generation of the Victoria Land Capability layer as they were deemed to be informative, and spatial data was available for use from data.vic.gov.au. Parameters highlighted in red may have been informative based on Victoria Land Capability Assessment or other states data use, but spatial data for which was not readily available from data.vic.gov.au. Where informative parameters did not have relevant spatial data, efforts were made to include similar parameters as much as possible. For example, while spatial data for 'soil depth' was not available, data for 'structure of subsoil' was deemed to be a suitable replacement.

Table S2 outlines the methods undertaken to produce the Victorian land capability layer, including transformation of input data and weighted combination of relevant data layers.

Table S1: Data Selection for Victorian land capability mapping

Parameter Name	From Assessment	Included in Layer	Data reference
**Soil type**		**Y**	Department of Economic Development, Jobs, Transport and Resources. Victorian Soil Type Mapping. [map]. [1:100,000]. Updated Jan 2018. https://www.data.vic.gov.au/data/dataset/victorian-soil-type-mapping
Soil depth	Y	N	NA
**Structure of subsoil**		**Y**	Department of Environment, Land, Water & Planning. Land Systems of Victoria at 1:250 000. [map]. [1:250,000]. Updated Feb 2018. DOI 10.4226/92/58e727e0dd1be
**Water erosion**	**Y**	**Y**	Department of Environment, Land, Water & Planning. Land Systems of Victoria at 1:250 000. [map]. [1:250,000]. Updated Feb 2018. DOI 10.4226/92/58e727e0dd1be
**Wind erosion**	**Y**	**Y**	Department of Environment, Land, Water & Planning. Land Systems of Victoria at 1:250 000. [map]. [1:250,000]. Updated Feb 2018. DOI 10.4226/92/58e727e0dd1be
Gully erosion	Y	N	NA
Surface rockiness	Y	N	NA
**Surface texture**	**Y**	**Y**	Department of Environment, Land, Water & Planning. Land Systems of Victoria at 1:250 000. [map]. [1:250,000]. Updated Feb 2018. DOI 10.4226/92/58e727e0dd1be
Physical cond. of surface soil	Y	N	NA
**Alkalinity**		**Y**	Department of Economic Development, Jobs, Transport and Resources. Victorian Soil pH mapping (VicDSMv1). [map]. [1:100,000]. Updated Jan 2018. https://www.data.vic.gov.au/data/dataset/victorian-soil-ph-mapping-vicdsmv1

**Acidity**		**Y**	Department of Economic Development, Jobs, Transport and Resources. Victorian Soil pH mapping (VicDSMv1). [map]. [1:100,000]. Updated Jan 2018. https://www.data.vic.gov.au/data/dataset/victorian-soil-ph-mapping-vicdsmv1
**Salinity (non-water table)**	**Y**	**Y**	Department of Environment, Land, Water & Planning. Land Systems of Victoria at 1:250 000. [map]. [1:250,000]. Updated Feb 2018. DOI 10.4226/92/58e727e0dd1be
**Waterlogging**	**Y**	**Y**	Department of Environment, Land, Water & Planning. Land Systems of Victoria at 1:250 000. [map]. [1:250,000]. Updated Feb 2018. DOI 10.4226/92/58e727e0dd1be
Total water availability	Y	N	NA
**Precipitation**	**Y**	**Y**	Department of Environment, Land, Water & Planning. Land Systems of Victoria at 1:250 000. [map]. [1:250,000]. Updated Feb 2018. DOI 10.4226/92/58e727e0dd1be
**Slope**	**Y**	**Y**	Department of Environment, Land, Water & Planning. Vicmap Elevation DTM 10m. [map]. [12.5m verticle, 5m horizontal]. Updated Jan 2018. https://www.data.vic.gov.au/data/dataset/vicmap-elevation-dtm-10m
Growing season length	Y	N	NA

Table S2: Data types, transformations, and uses to compile the final Victorian land capability data

Data	Data Type	Transformation	Outcome	Use	Final
**Soil Type**	Shapefile storing information about soil type.	Shapefile converted to raster and reclassified to numerical rating.	Rating based on soil attributes described by Australian Soil Club (http://www.soil.org.au/soil-types.htm).	All soil layers were combined and averaged to develop a soil suitability layer.	Soil suitability, slope suitability, and rainfall suitability layers combined in raster calculator and averaged to get a 'mean land suitability' layer. The 'mean land suitability' layer was then smoothed using majority filtering and reclassified in accordance with NSW rating scale.
**Soil Compaction Susceptibility**	Raster grid storing information about soil compaction.	Extracted from Victorian Land Systems shapefile layer by converting to a raster based on "COMP_SUSC". Reclassified to numerical rating.	Numerical rating scaled so that soils with higher compaction susceptibility scored lower than soils with low compaction.		
**Soil Salinity**	Raster grid storing information about soil salinity.	Extracted from Victorian Land Systems shapefile layer by converting to a raster based on "SALT". Reclassified to numerical rating.	Numerical rating scaled so that soils with higher soil salinity scored lower than soils with low salinity as per NSW Office of Environment and Heritage "The land and soil capability assessment scheme – second approximation".		
**Water Erosion Susceptibility**	Raster grid storing information about water erosion.	Extracted from Victorian Land Systems shapefile layer by converting to a raster based on "WATER_SUSC". Reclassified to numerical rating.	Numerical rating scaled so that soils with higher water erosion susceptibility scored lower than soils with low erosion in accordance with VRO Land Capability Assessment guidelines.		
**Waterlogging Susceptibility**	Raster grid storing information about waterlogging.	Extracted from Victorian Land Systems shapefile layer by converting to a raster based on "WLOG_SUSC". Reclassified to numerical rating.	Numerical rating scaled so that soils with higher waterlogging susceptibility scored lower than soils with low waterlogging.		
**Wind Erosion Susceptibility**	Raster grid storing information about wind erosion.	Extracted from Victorian Land Systems shapefile layer by converting to a raster based on "WIND_SUSC". Reclassified to numerical rating.	Numerical rating scaled so that soils with higher wind erosion susceptibility scored lower than soils with low wind erosion in accordance with VRO Land Capability Assessment guidelines.		
**Soil pH**	Shapefile storing information about soil pH.	Shapefile converted to raster and reclassified to numerical rating in accordance with VRO Land Capability Assessment guidelines	Numerical rating scaled so that more alkaline soils and more acidic soils scored lower than soils roughly neutral as per QLD Regional Land Suitability Framework.		
**Slope**	DEM of Victoria (tif).	DEM converted to slope.	Slope reclassified to a numerical rating in accordance with VRO Land Capability Assessment guidelines.	Slope suitability layer.	
**Rainfall**	Raster grid storing information about rainfall.	Extracted from Victorian Land Systems shapefile layer by converting to a raster based on "CLIMATE". Reclassified to numerical rating.	Numerical rating scaled so that more rain was better up until >700mm where ratings stayed the same.	Rainfall suitability layer.	

Northern Territory detailed methods:

Pre-prepared Land Capability maps were available for different agricultural land classes in the norther region of the Northern Territory and for Southern Rangelands [3]. These Land Capability maps were reclassified using stored information and converted to the NSW rating scheme. Pastoral potential maps for the whole state were also available, however as they were binary (either suitable or unsuitable with no scaling) they were not used and the pastoral potential gap fill layer was used instead. In the table below (Table S3) we detail the steps used to compile the final Northern Territory land capability data for the agricultural regions.

Table S3: Data types, transformations, and uses to compile the final Northern Territory land capability data

Data	Data Type	Transformation	Outcome	Use	Final
**Northern Region Field Crops**	Shapefile clipped to the extent of land suitability for field crops. Stores information about the land capability.	Information about suitability (erosion risk, acid-sulphate soil occurrence, soil quality, irrigation potential) contained in shapefile extracted, converted to raster, and combined to numerically rank capability of different regions.	Numerical raster with high values indicating areas with higher suitability for field cropping.	Layer was reclassified based on NSW classification scheme. Field crop layer was reclassified to a 3 – 5 scale (3 = high suitability, 4 = medium suitability, 5 = low suitability).	Land Capability layers were mosaicked together selecting the highest score possible (minimum value) for each grid cell. This method allows for the retention of areas of land that score high for intensive uses (eg perennial horticulture) but potentially score low for less intense uses (eg grazing).
**Northern Region Annual Horticulture**	Shapefile clipped to the extent of land suitability for annual horticulture. Stores information about the land capability.	Information about suitability (erosion risk, acid-sulphate soil occurrence, soil quality, irrigation potential) contained in shapefile extracted, converted to raster, and combined to numerically rank capability of different regions.	Numerical raster with high values indicating areas with higher suitability for annual horticulture.	Layer was reclassified based on NSW classification scheme. Horticulture layers were reclassified to a 2 – 5 scale (2 = very high suitability, 3 = high suitability, 4 = medium suitability, 5 = low suitability).	
**Northern Region Perennial Horticulture**	Shapefile clipped to the extent of land suitability for perennial horticulture. Stores information about the land capability.	Information about suitability (erosion risk, acid-sulphate soil occurrence, soil quality, irrigation potential) contained in shapefile extracted, converted to raster, and combined to numerically rank capability of different regions.	Numerical raster with high values indicating areas with higher suitability for perennial horticulture.	Layer was reclassified based on NSW classification scheme. Horticulture layers were reclassified to a 2 – 5 scale (2 = very high suitability, 3 = high suitability, 4 = medium suitability, 5 = low suitability).	
**Southern Region Agricultural Suitability**	Shapefile clipped to the extent of land suitability for agriculture broadly (in Southern region). Stores information about the land capability.	Agricultural suitability description contained in shapefile extracted and converted to raster to numerically rank capability of different regions.	Layer was reclassified based on NSW classification scheme. Southern region agriculture layers were reclassified to a 4 – 5 scale (4 = high suitability, 5 = medium/low suitability). No areas of the southern rangelands were deemed suitable to score above a 4 due to water/land limitations.		

Western Australia detailed methods:

Pre-prepared Land Capability maps were available for different agricultural land classes in the south-western region of Western Australia. These Land Capability maps were reclassified using ranking information and converted to the NSW rating scheme. Pastoral potential maps for specific rangelands were also available, and the pastoral potential gap fill layer was used where gaps existed in this data. In the table below (Table S4) we detail the steps used to compile the final Western Australian land capability data for the agricultural region.

Table S4: Data types, transformations, and uses to compile the final Western Australia land capability data

Data	Data Type	Transformation	Outcome	Use	Final
**WA Land Capability – Annual Horticulture**	Shapefile clipped to the extent of lands mapped for agricultural capability in Western Australia (wheatbelt region). Shapefile ranked land for capability to support annual horticulture.	Shapefile converted to raster with categorical rating scheme converted to numerical ranks.	Numerical raster with higher values indicating high capability to support annual horticulture.	Layer was reclassified based on NSW classification scheme. Annual horticulture was reclassified to 2 – 5 scale (2 = very high, 3 = high, 4 = medium, 5 = low).	Land Capability layers were mosaicked together selecting the highest rank possible (minimum value) for each grid cell. This method allows for the retention of areas of land that score high for intensive uses (eg perennial horticulture) but potentially score low for less intense uses (eg grazing).
**WA Land Capability – Perennial Horticulture**	Shapefile clipped to the extent of lands mapped for agricultural capability in Western Australia (wheatbelt region). Shapefile ranked land for capability to support annual horticulture.	Shapefile converted to raster with categorical rating scheme converted to numerical ranks.	Numerical raster with higher values indicating high capability to support perennial horticulture.	Layer was reclassified based on NSW classification scheme. Perennial horticulture was reclassified to 2 – 5 scale (2 = very high, 3 = high, 4 = medium, 5 = low).	
**WA Land Capability – Dryland Cropping**	Shapefile clipped to the extent of lands mapped for agricultural capability in Western Australia (wheatbelt region). Shapefile ranked land for capability to support annual horticulture.	Shapefile converted to raster with categorical rating scheme converted to numerical ranks.	Numerical raster with higher values indicating high capability to support dryland cropping	Layer was reclassified based on NSW classification scheme. Dryland cropping was reclassified to 3 – 5 scale (3 = high, 4 = medium, 5 = low).	
**WA Land Capability – Vineyards**	Shapefile clipped to the extent of lands mapped for agricultural capability in Western Australia (wheatbelt region). Shapefile ranked land for capability to support annual horticulture.	Shapefile converted to raster with categorical rating scheme converted to numerical ranks.	Numerical raster with higher values indicating high capability to support vineyards.	Layer was reclassified based on NSW classification scheme. Vineyards was reclassified to 3 – 5 scale (3 = high, 4 = medium, 5 = low).	
**WA Land Capability - Grazing**	Shapefile clipped to the extent of lands mapped for agricultural capability in Western Australia (wheatbelt region). Shapefile ranked land for capability to support annual horticulture.	Shapefile converted to raster with categorical rating scheme converted to numerical ranks.	Numerical raster with higher values indicating high capability to support grazing.	Layer was reclassified based on NSW classification scheme. Grazing was reclassified to 4 – 7 scale (4 = very high, 5 = high, 6 = medium, 7 = low).	

South Australia detailed methods:

Pre-prepared Land Capability maps were available for specific crop types for different agricultural land classes in the southern region of South Australia. These crop-specific Land Capability maps were averaged by land class and converted to the NSW rating scheme. Pastoral potential maps were not available, hence the pastoral potential gap fill layer was used. In the table below (Table S5) we detail the steps used to compile the final South Australia land capability data for the agricultural region.

Table S5: Data types, transformations, and uses to compile the final South Australia land capability data

Data	Data Type	Transformation	Outcome	Use	Final
**Annual Horticulture**	Shapefiles for various annual horticulture crops clipped to the extent of lands mapped for agricultural capability in South Australia. Shapefile ranked land for capability to support annual horticulture.	Each individual shapefile was converted to raster with categorical rating scheme converted to numerical ranks. Rasters were combined to get the average rank for various annual horticulture species at each cell.	Numerical raster with higher values indicating high average capability to support annual horticulture.	Layer was reclassified based on NSW classification scheme. Annual horticulture was reclassified to 2 – 5 scale (2 = very high, 3 = high, 4 = medium, 5 = low).	Land Capability layers were mosaicked together selecting the highest rank possible (minimum value) for each grid cell. This method allows for the retention of areas of land that score high for intensive uses (eg perennial horticulture) but potentially score low for less intense uses (eg grazing).
**Perennial Horticulture**	Shapefiles for various perennial horticulture crops clipped to the extent of lands mapped for agricultural capability in South Australia. Shapefile ranked land for capability to support annual horticulture.	Each individual shapefile was converted to raster with categorical rating scheme converted to numerical ranks. Rasters were combined to get the average rank for various perennial horticulture species at each cell.	Numerical raster with higher values indicating high average capability to support perennial horticulture.	Layer was reclassified based on NSW classification scheme. Perennial horticulture was reclassified to 2 – 5 scale (2 = very high, 3 = high, 4 = medium, 5 = low).	
**Field Crops**	Shapefiles for various field crops clipped to the extent of lands mapped for agricultural capability in South Australia. Shapefile ranked land for capability to support annual horticulture.	Each individual shapefile was converted to raster with categorical rating scheme converted to numerical ranks. Rasters were combined to get the average rank for various field crop species at each cell.	Numerical raster with higher values indicating high average capability to support field cropping.	Layer was reclassified based on NSW classification scheme. Field crops was reclassified to 2 – 5 scale (2 = very high, 3 = high, 4 = medium, 5 = low).	
**Dryland Pasture**	Shapefiles for various dryland pasture crops clipped to the extent of lands mapped for agricultural capability in South Australia. Shapefile ranked land for capability to support annual horticulture.	Each individual shapefile was converted to raster with categorical rating scheme converted to numerical ranks. Rasters were combined to get the average rank for various dryland pasture species at each cell.	Numerical raster with higher values indicating high average capability to support dryland pasture.	Layer was reclassified based on NSW classification scheme. Dryland pasture was reclassified to 3 – 6 scale (3 = very high, 4 = high, 5 = medium, 6 = low).	
**Irrigated Pasture**	Shapefiles for various irrigated pasture crops clipped to the extent of lands mapped for agricultural capability in South Australia. Shapefile ranked land for capability to support annual horticulture.	Each individual shapefile was converted to raster with categorical rating scheme converted to numerical ranks. Rasters were combined to get the average rank for various irrigated pasture species at each cell.	Numerical raster with higher values indicating high average capability to support irrigated pasture.	Layer was reclassified based on NSW classification scheme. Irrigated pasture was reclassified to 4 – 6 scale (4 = high, 5 = medium, 6 = low).	

References

[1] OEH, The land and soil capability assessment scheme: Second approximation, State of NSW and Office of Environment and Heritage (OEH), Sydney, NSW, 2012.

[2] R. Rowe, D. Howe, N. Alley, Guidelines for Land Capability Assessment in Victoria, Soil Conservation Authority, Kew, Victoria, 1981.

[3] A. Pascoe-Bell, B. Lynch, J. Hill, C. Green, A. Cameron, S. Smith, Identification of potential land for long-term sustainable food production, Department of Natural Resources, Environment, The Arts and Sport (NRETAS), Palmerston, Northern Territory, 2011.
